# THE IMPACT OF MANDATED COGNITIVE ASSESSMENTS ON ALZHEIMER’S DISEASE AND RELATED DEMENTIA DIAGNOSIS RATES

**DOI:** 10.1093/geroni/igz038.3055

**Published:** 2019-11-08

**Authors:** Johanna Thunell, Julie Zissimopoulos

**Affiliations:** University of Southern California, Los Angeles, California, United States

## Abstract

Although no cure for Alzheimer’s disease exists, early diagnosis allows clinicians to detect reversible causes of memory loss, inform pharmacologic treatment options that may delay cognitive decline, and inform patients about clinical trial opportunities. It allows patients to communicate medical, legal, financial, living, and end-of-life desires. Barriers to diagnosis include low public awareness of early symptoms, stigma and misconceptions about the disease that delay seeking medical assistance. Provider-related barriers include low recognition of cognitive impairment and/or insufficient training in dementia diagnosis, and reimbursement issues. The new annual wellness visit (AWV) benefit available to Medicare Part B beneficiaries may reduce some of these barriers. The Patient Protection and Affordable Care Act of 2010 mandated an AWV that, along with routine preventive services, included for the first time, a cognitive screen at each visit. In this study, we analyze the effect of the introduction of the AWV benefit on Alzheimer’s disease and related dementias (ADRD) diagnoses rates, average age at diagnosis and geographic dispersion of diagnoses. We use the 100% sample of Medicare claims and regression discontinuity to estimate the impact of the legislation enactment. Preliminary analyses show immediate and increasing take-up in AWV visits after the policy went into effect. The total number of preventative exams claims went from about 2,500 to 27,000 immediately after the policy and continued to grow in subsequent months. We also observe an increase in ADRD incidence in the months immediately following ACA, from about 14,000 claims in December 2010 to about 16,000 in January 2011.

